# Standardization and Validation of Digital Volumetric Measurement Methods for Alveolar Cleft Defects Using 3D Imaging

**DOI:** 10.3390/dj14050247

**Published:** 2026-04-23

**Authors:** Inka Saraswati, Menik Priaminiarti, Dwi Ariawan, Sariesendy Sumardi, Bramma Kiswanjaya, Bayu Trinanda Putra, Hanna H. Bachtiar-Iskandar, Norifumi Nakamura, Muhammad Syafrudin Hak, Heru Suhartanto, Takeshi Mitsuyasu

**Affiliations:** 1 Department of Dentomaxillofacial Radiology, Faculty of Dentistry, Universitas Indonesia, Salemba Raya No. 4, Central Jakarta 10430, Indonesia; inkasaraswati@office.ui.ac.id (I.S.); bramma.kiswanjaya@ui.ac.id (B.K.); bayu.trinanda@ui.ac.id (B.T.P.);; 2 Department of Oral and Maxillofacial Surgery, Faculty of Dentistry, Universitas Indonesia, Salemba Raya No. 4, Central Jakarta 10430, Indonesia; dwi.ariawan02@ui.ac.id (D.A.); gigi1394nakamura@gmail.com (N.N.); 3 Department of Orthodontics, Faculty of Dentistry, Universitas Indonesia, Salemba Raya No. 4, Central Jakarta 10430, Indonesia; sariesendy.sumardi@ui.ac.id; 4 Department of Oral and Maxillofacial Surgery, Graduate School of Dental and Medical Sciences, Kagoshima University, 8-35-1, Sakuragaoka, Kagoshima 890-8544, Japan; 5 Cleft Center, Harapan Kita Children and Mother Hospital, Letnan Jenderal S Parman Kavling. 87, West Jakarta 11420, Indonesia; 6 Department of Oral and Maxillofacial Surgery, Faculty of Dentistry, Universitas Padjajaran, Jatinangor 45363, Indonesia; 7 Faculty of Computer Science, Universitas Indonesia, Jalan. Lingkar Pondok Cina, Depok 16424, Indonesia; heru@cs.ui.ac.id; 8 Division of Maxillofacial Diagnostic and Surgical Science, Faculty of Dental Science, Kyushu University, 3-1-1 Maidashi, Higashi-ku, Fukuoka 812-8582, Japan

**Keywords:** bone grafting, CBCT, computed tomography, 3D imaging, cleft lip, cleft palate

## Abstract

**Background/Objectives:** Accurate quantification of alveolar cleft defects for bone grafting remains difficult due to inconsistent anatomical boundaries. This study established an expert consensus on boundary landmarks for alveolar bone graft (ABG) planning and validated the accuracy and reliability of digital volumetric measurement methods. **Methods:** Three cleft specialists performed repeated simulated graft procedures in seven patient-specific 3D-printed models, first according to the operator’s clinical judgment, and subsequently according to panel-derived consensus boundaries. Two radiologists independently conducted digital volumetric assessments in 3D X-ray imaging using four measurement approaches (axial tracing, interpolated axial tracing, landmark-based mirroring, and mesh-based mirroring), generating 56 independent digital segmentations to be evaluated against the consensus-based physical reference standard. Volumes of the defects were recorded, intra- and inter-rater reliabilities were calculated using the intraclass correlation coefficient (ICC), and differences among methods were analyzed. **Results:** Operator-defined plans showed significant inter-operator differences (*p* < 0.001) with poor-to-excellent reliability (intra-rater ICC 0.060–0.967; inter-rater ICC 0.300–0.635). Consensus established standardized boundaries: tilted plane from base of anterior nasal spine to hard palate, cemento-enamel junctions, incisive canal, and alveolar contour. Consensus-based filling showed non-significant inter-rater differences (*p* = 0.139) and substantially improved reliability (intra-rater ICC 0.904–0.988; inter-rater ICC 0.622–0.861). Among the four digital methods evaluated, axial tracing demonstrated excellent reliability (intra-rater ICC 0.971–0.99; inter-rater ICC 0.965) and high accuracy (mean difference 0.001–0.026 cm^3^), with no significant difference (*p* = 0.999) from the physical reference standard. **Conclusions:** These proposed consensus-based boundary definitions and validated volumetric measurement methods improved the accuracy and reproducibility of personalized alveolar bone graft planning.

## 1. Introduction

The alveolar cleft represents a bony discontinuity observed in approximately 75% of individuals with cleft lip and palate [1]. Alveolar bone grafting is crucial for cleft patients with alveolar involvement, as it supports tooth eruption, maintains periodontal health, facilitates orthodontic treatment, prepares for implant placement, aids in fistula closure, and prepares for orthognathic surgery. Volumetric measurements of alveolar cleft defects have been increasingly adopted to improve planning for bone graft surgeries, more precisely assess resorption rates, identify predictors of success, and evaluate novel bone grafting materials [2,3]. Key outcome metrics, such as bone fill or resorption percentage, rely on accurate comparisons of postoperative bone volume to preoperative defect.

The widespread adoption of three-dimensional dental imaging has led to the broader use of volumetric evaluations in clinical care and research, including in cleft care. Measuring postoperative bone volume is reasonably straightforward when 3D image quality is adequate. In contrast, quantifying the preoperative defect for alveolar bone graft planning is considerably more complex due to the absence of anatomical boundaries on the palatal, buccal, occlusal, and nasal sides of the defect. One of the most crucial aspects in measuring cleft defect for alveolar bone graft planning is determining the boundaries of the expected graft filling, which directly relate to the quantified volume. According to a study by Wang et al. [4], the volume measured with the ANS–PNS upper boundary differs significantly from measurement using the ANS–GPF. A recent systematic review found substantial variability in the landmarks used to define defect boundaries across studies. In most studies, landmark descriptions were inadequate or absent [5]. Currently, there is limited comparability between studies using preoperative alveolar defect volume due to heterogeneity in boundary descriptions. Standardized landmark-defined boundaries would greatly improve comparability between studies and promote more reliable surgical planning in daily practice.

The review also noted that multiple measurement methods were described in the literature, ranging from as simple as geometric multiplication to as advanced as artificial intelligence (AI). Very few studies contained head-to-head comparisons between several digital volumetric measurement methods, limiting the ability to identify the optimal approach. Volumetric assessment of the alveolar cleft will likely become more widespread in the future with the maturation of AI tools. All approaches, with or without AI, should be developed based on empirical evidence. Therefore, research directly comparing multiple methods is required to determine the optimal measurement method.

There are many aspects that may contribute to differences between surgeons in alveolar bone graft surgery, such as experience [6], surgical technique [7], general skill [8], case selection [6,9], institution-wide protocol (for example, pre-surgery or post-surgery orthodontics, timing, etc.) [10], and choice of graft material or donor site [11]. Those aspects are beyond the scope of the current study, but the amount of bone graft inserted into the defect remains one of the most defining factors of determining how much bone bridge will form. Under-filling and over-filling has been observed in the literature [12,13,14]. Different surgeons have also been documented to insert different amounts of filling material [15]. Therefore, standardized and explicit boundary definitions, combined with preoperative volumetric planning, should help improve consistency between surgeons and promote more reliable outcomes.

This study aims to move forward from knowledge synthesis to preclinical validation of various volumetric alveolar cleft measurement methods. This step is intended to support the development of pre-operative planning protocol and evaluation of alveolar bone graft surgery to reduce between-study variability and improve surgical planning.

Three specific objectives were formulated: quantitative investigation of an operator-specified plan; development of a consensus-derived boundary definition for idealized alveolar bone graft filling; quantitative and qualitative validation for accuracy and reliability of various digital measurement methods against the physical reference standard.

## 2. Materials and Methods

The study was conducted from December 2024 to October 2025 using secondary preoperative 3D imaging data. Samples were consecutively collected as secondary data from seven patients with unilateral alveolar and palatal clefts who were scheduled for alveolar bone graft surgery between March and December 2024 at Harapan Kita Mother and Child Hospital, Jakarta, Indonesia. Sample size was calculated for α = 0.05, 90% power, and 300 mm^3^ standard deviation, based on previous study [13], and a desired difference of 365 mm^3^. Prior to commencing the study, four patients (57%) were randomly chosen from the samples for intra-observer tests. The same patients were used consistently for intra-observer assessments throughout all sections of the study. This study was reviewed and received ethical approval (57/Ethical Approval/FKGUI/IX/2024 on 3 September 2024) from the Faculty of Dentistry, Universitas Indonesia, and was conducted in compliance with the Declaration of Helsinki. Patient consent was waived due to the use of secondary data.

Inclusion criteria were unilateral cleft lip and palate with an accompanying alveolar defect, planned secondary alveolar bone graft surgery, and available CT or CBCT imaging obtained within 6 months before surgery. In addition, the DICOM data had to be successfully retrievable. Exclusion criteria included syndromic cleft lip and/or palate, prior bone grafting of the alveolar defect, and any previous surgical intervention that could have altered morphology of the graft site. Cases were also excluded if image quality was insufficient for accurate segmentation.

Measurement performance was evaluated in terms of accuracy and reliability. Accuracy was defined as agreement between each digital method and the physical reference standard, whereas reliability was defined as inter- and intra-observer consistency. Accuracy was assessed using mean differences with 95% confidence intervals and repeated-measures analysis, while reliability was assessed using intraclass correlation coefficients (ICCs) for absolute agreement.

### 2.1. Data and Model Preparation

Imaging data taken was computed tomography (CT) or cone-beam computed tomography (CBCT) from multiple sources due to the absence of an in-house CBCT. To standardize the dataset, images were resampled to a 0.250 mm isotropic voxel size and normalized to a 0–4095 gray value. Skulls were re-oriented until the anterior nasal spine (ANS)–posterior nasal spine (PNS) and right–left major palatine foramen were horizontally coplanar to each other.

DICOM datasets were imported into Slicer (www.slicer.org) to perform automatic segmentation of dentition, maxilla, facial, and cranial bones using Dental Segmentator extension. Manual segmentation refinement in the alveolar cleft area was subsequently carried out with paint and erase tools to ensure accuracy. The same segmentations were used for both digital and 3D-printed evaluations. All digitally derived volumes were determined via segmentation voxel count.

In order to prepare for 3D printing, the segmentations were cropped and converted into STL files with Slicer. A 10.00 mm block was added to each model’s base to allow for measuring model size deviation using the digital caliper Digimatic CD-6” CSX (Mitutoyo, Kawasaki, Japan). PETG FDM models were 3D-printed with Bambu Lab P1S (Bambu Lab, Shenzhen, China). Printing parameters were 0.2 mm nozzle, 0.2 mm layer height, three bottom shell layers, five top shell layers, and 20% grid infill [16,17]. Each patient’s models were reused for all relevant parts of the studies. Non-drying clay material Jolly King Plasteline (Chavant Clay, Farmingdale, NY, USA) was used for the simulated graft. The simulation material was selected for its dimensional stability and handling properties. It also showed agreement with measurements obtained using a conventional water displacement method. After filling of the alveolar defects, the clay material was carefully removed and weighed using a Shimadzu AX200 (Shimadzu, Kyoto, Japan) analytical balance with 0.0001 g readability. Each simulated graft was weighed twice. The clay material had a known density of 102.3 lbs/cu.ft (1.638688764 gr/cm^3^) based on the manufacturer’s information, therefore, the volume of the simulated graft was calculated by dividing the clay’s average weight by its density.

### 2.2. Quantitative Evaluation of Specialists’ Plans

Two surgeons (Rater 1: 39 years as a maxillofacial surgeon, including 30 years in cleft surgery; Rater 2: 16 years as a maxillofacial surgeon, including 8 years in cleft surgery) and one orthodontist (Rater 3: 18 years as an orthodontist, including 10 years in cleft care) were chosen as raters due to their role in performing alveolar bone graft surgery and assessing the outcome of alveolar bone graft for subsequent treatment, respectively. They all specialized in cleft management. They manually filled alveolar defects in 3D-printed models using clay material as a simulation of alveolar bone graft filling. All raters were instructed to fill the models according to their personal professional judgment. Each rater was blinded and filled the models with at least a one-month interval for intra-observer measurements.

The volume of each simulated graft was obtained. Statistical comparison between each rater was conducted using repeated measures ANOVA followed by Bonferroni post hoc analysis. Values of *p* < 0.05 were considered significant, and the mean difference and 95% CI for difference were reported. ICCs for absolute agreement were calculated for inter- and intra-rater reliability. ICC values of >0.90 were considered excellent agreement, 0.75 to <0.90 were good agreement, 0.5 to <0.75 were moderate agreement, and <0.5 were poor agreement.

### 2.3. Development of a Consensus-Derived Boundary Definition for Idealized Alveolar Bone Graft Filling

**Figure 1:**
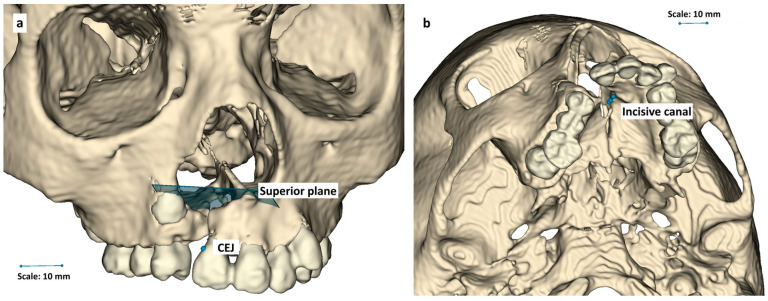
Landmarks for alveolar cleft boundary definitions from a (**a**) frontal and (**b**) occlusal view.

After investigating operator-specified plans in accordance with the first objective, a definitive description of landmarks for graft boundaries was sought. Based on systematic review [5] and a consensus between aforementioned cleft experts and a radiologist (6 years of experience), the following anatomical landmarks (Figure 1) were carefully chosen:

• Upper boundary: A tilted plane connecting the base of the anterior nasal spine (ANS) to the upper surface of the hard palate on the cleft side.
• Lower boundary: The cemento-enamel junctions (CEJs).
• Lateral boundary: Contiguous with adjacent hard tissues.
• Buccal contour: Symmetrical with the contralateral side, creating smooth continuity between segments.
• Palatal contour of the alveolar bone: Symmetrical with the contralateral side, creating smooth continuity between segments.
• In cases involving the palate, the superior palatal boundary line passes through the anterior edge of the incisive canal.

These boundaries were designed to represent the idealized volume and shape of alveolar bone graft material insertion without under- or over-filling. The boundaries were formulated to be relatively constant and relevant both intra-operatively and in imaging. Patient-specific and case-specific factors that could influence graft volume were not considered, such as soft tissue availability, scarring, nasal concha position, bone graft material, etc.

After calibration to standardize interpretation of the predefined anatomical boundaries, the same three raters were instructed to simulate graft filling in the 3D-printed models according to the newly devised boundaries, repeated after at least a one-month interval for intra-rater measurements. All raters were blinded, and the volume of each simulated graft was obtained. Statistical comparison between each rater was conducted using repeated measures ANOVA followed by Bonferroni post hoc analysis. Additionally, paired t-tests were used to analyze the differences against the same operator’s specific plan. Values of *p* < 0.05 were considered significant, and the mean difference and 95% CI for difference were reported. ICCs for absolute agreement were calculated for inter- and intra-rater reliability. ICC values of >0.90 were considered excellent agreement, 0.75 to <0.90 were good agreement, 0.5 to <0.75 were moderate agreement, and <0.5 were poor agreement.

### 2.4. Quantitative and Qualitative Validation of Various Digital Measurement Methods Against 3D-Printed Models

Two radiology experts (Rater 1: 6 years of experience; Rater 2: 4 years of experience) underwent training and calibration by an experienced radiologist proficient in Slicer software (v.5.8.1) according to a standardized operating procedure. After calibration, the raters were blinded and independently performed all digital defect segmentations and measurements. Four digital volumetric measurement methods were compared against the average aforementioned consensus-based volume. A minimum two-week interval was maintained between each application of the four methods, as well as between repeated measurements for intra-observer assessment. 3D reconstructions and multiplanar imaging slices were also reviewed to qualitatively assess the restored anatomy and the adequacy of the graft.

The digital measurement methods were as follows:

• Method A: Manual tracing in every axial slice.
• Method B: Manual tracing in every fifth slice (1.25 mm interval), followed by automatic interpolation.
• Method C: Mirroring patient’s skull, followed by landmark-based superimposition.
• Method D: Mirroring patient’s skull, followed by mesh-based superimposition.

These methods were appointed based on previous systematic reviews [5,18]. Methods A and B followed boundary lines established in this study. Method B was a modification of the first method devised to reduce measurement time.

In Method C, superimposition was based on three anatomical landmarks: ANS, right major palatine foramen, and left major palatine foramen. Prior to performing Method D, segmentations were converted into STL files. The volume of interest in Method D was bounded by a plane 1 mm above the ANS superiorly, lowermost CEJ inferiorly, lowermost point of the infraorbital rim laterally, and the distalmost point of the maxillary first molars posteriorly. After superimposition in Methods C and D, the original maxilla was subtracted from the mirrored maxilla. These superimposition landmarks were selected to maximize the superimposition of the alveolar area without influencing the dentition or larger maxillary structures. Manual refinement using the scissors tool in Methods C and D was conducted to crop out structures beyond the defect area bounded by ANS, CEJ, the incisive canal, and the most prominent bone contour. Voids within cancellous areas were manually filled using the paint tool to ensure that the segmented defects formed a solid object, thereby allowing accurate volume calculation.

The volume of each virtual graft was obtained. ICCs were calculated for inter- and intra-rater reliability. Simulated graft volumes obtained from 3D-printed models in the previous section were averaged to act as a reference standard, then compared with the average volume from each method using repeated measures ANOVA followed by Bonferroni post hoc analysis. Values of *p* < 0.05 were considered significant, and the mean difference and 95% CI for difference were reported.

## 3. Results

Seven patients were included in this study after reviewing inclusion and exclusion criteria. Five patients were males (71%) and two were females (29%). The mean age was 11.85 ± 2.4. Mean deviation of the 3D-printed model’s accuracy was 0.04 ± 0.0098 mm, ranging from 0.02 to 0.05 mm.

### 3.1. Exploration of Operator-Specified Plan

**Figure 2:**
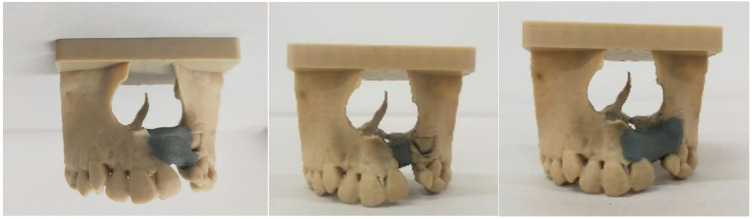
Operator-specified plans from raters 1–3 in 3D-printed models. Grey areas indicated simulated graft filling.

Mean defect volumes were 1.135 ± 0.240 cm^3^, 0.535 ± 0.100 cm^3^, and 0.890 ± 0.311 cm^3^ based on each specialist’s plan, respectively. Representative images can be seen in Figure 2. ANOVA showed a significant difference between raters (*p* = <0.001, η_p_^2^ = 0.761). Post hoc Bonferroni testing indicated that the first rater demonstrated a significantly higher mean volume than the second rater (*p* = <0.001, mean difference = 0.600 cm^3^, 95% CI [0.364, 0.836]). No other rater differences were significant (*p* = 0.172, mean difference = 0.245 cm^3^, 95% CI [−0.98, 0.587] and *p* = 0.058, mean difference = 0.355 cm^3^, 95% CI [−0.14, 0.724]). Intra-observer ICCs for raters 1–3 were 0.967 (excellent), 0.060 (poor), and 0.148 (poor), respectively. The inter-observer ICC between Rater 1 and 2 was 0.635 (moderate), between Rater 1 and 3 was 0.524 (moderate), and between Rater 2 and 3 was 0.300 (poor).

### 3.2. Consensus-Defined Alveolar Defect Volume

**Figure 3:**
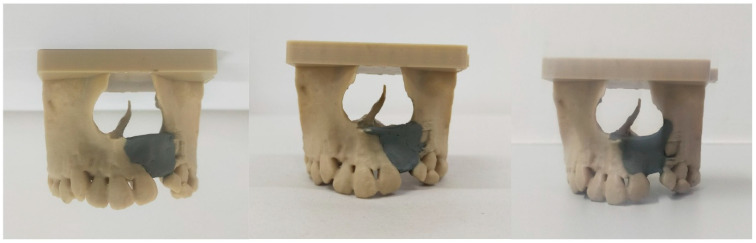
Consensus-based plans from raters 1–3 in 3D-printed models. Grey areas indicated simulated graft filling.

After applying consensus-derived boundaries, mean defect volumes for each rater were 1.136 ± 0.342 cm^3^, 1.243 ± 0.171 cm^3^, and 1.199 ± 0.256 cm^3^, respectively. Representative images can be seen in Figure 3. There was no significant difference between raters (*p* = 0.139, η_p_^2^ = 0.251). However, consensus-based volumes were significantly different from the corresponding rater’s specific plan (*p* = 0.042, Cohen’s d = 0.228, mean difference = 0.222 cm^3^, 95% CI [0.011, 0.432]; *p* = <0.001, Cohen’s d = 0.115, mean difference = 0.708 cm^3^, 95% CI [0.601, 0.814]; *p* = 0.041, Cohen’s d = 0.314, mean difference = 0.308 cm^3^, 95% CI [0.018, 0.598]). Intra-observer ICCs for raters 1–3 were 0.965 (excellent), 0.988 (excellent), and 0.904 (excellent), respectively. The inter-observer ICC between Rater 1 and 2 was 0.861 (good), between Rater 1 and 3 was 0.779 (good), and between Rater 2 and 3 was 0.622 (moderate).

### 3.3. Quantitative and Qualitative Validation of Various Digital Measurement Methods Against 3D-Printed Models

In this section of the study, two radiology experts conducted alveolar cleft measurements using four different methods, which were compared to a reference standard. The approximate time required for segmentation was 45 min for Method A, 20 min for Method B, 40 min for Method C, and 25 min for Method D. The reference standard was the average volume derived from clay-filling simulations based on specialist consensus as described in the previous section. Mean defect volumes and reliability values are presented in Table 1.

ANOVA between Rater 1 (Methods A–D) and the reference standard showed no significant difference (*p* = 0.619, η_p_^2^ = 0.101). Based on the Bonferroni post hoc test, measurement accuracy by Rater 1 from highest to lowest was Method C, B, A, then D (Method C *p* = 0.999, mean difference = 0.007 cm^3^, 95% CI [−0.337, 0.323]; Method B *p* = 0.999, mean difference = 0.009 cm^3^, 95% CI [−0.188, 0.207]; Method A *p* = 0.999, mean difference = 0.026 cm^3^, 95% CI [−0.278, 0.330]; Method D *p* = 0.999, mean difference = 0.113 cm^3^, 95% CI [−0.321, 0.547]).

For Rater 2, ANOVA showed a statistically significant difference between Methods A–D and the reference standard, with a large effect size (*p* = 0.001, η_p_^2^ = 0.514). However, Bonferroni-adjusted post hoc testing showed no significant pairwise differences (*p*-values ranging 0.068–1.000). This was likely related to the conservative nature of Bonferroni adjustment when multiple groups are compared. Based on the Bonferroni post hoc test, measurement accuracy by Rater 2 in descending order was Method A, B, D, then C (Method A *p* = 0.999, mean difference = 0.001 cm^3^, 95% CI [−0.257, 0.258]; Method B *p* = 0.999, mean difference = 0.007 cm^3^, 95% CI [−0.139, 0.152]; Method D *p* = 0.568, mean difference = 0.292 cm^3^, 95% CI [−0.243, 0.827]; Method C *p* = 0.068, mean difference = 0.478 cm^3^, 95% CI [−0.033, 0.988]). Due to conflicting ANOVA and Bonferroni post hoc test results, an exploratory re-analysis with a less conservative pairwise comparison (LSD) was conducted which revealed significant differences in Rater 2 when Method C was compared to the reference standard (*p* = 0.007, mean difference = 0.478 cm^3^, 95% CI [0.188, 0.767]), Method A (*p* = 0.017, mean difference = 0.478 cm^3^, 95% CI [0.120, 0.836]), and Method B (*p* = 0.013, mean difference = 0.471 cm^3^, 95% CI [0.139, 0.803]). Given that the LSD test is more sensitive than the Bonferroni test, this suggests definitive compatibility between Methods A, B, and D with the reference standard due to their non-significance. For Method C, the possibility of a true difference as detected with ANOVA cannot be excluded in this small dataset, but such difference may have been attenuated by the more conservative correction. Nevertheless, this did not alter the overall interpretation, because Method C was also judged severely deficient based on reliability and qualitative analysis.

**Figure 4:**
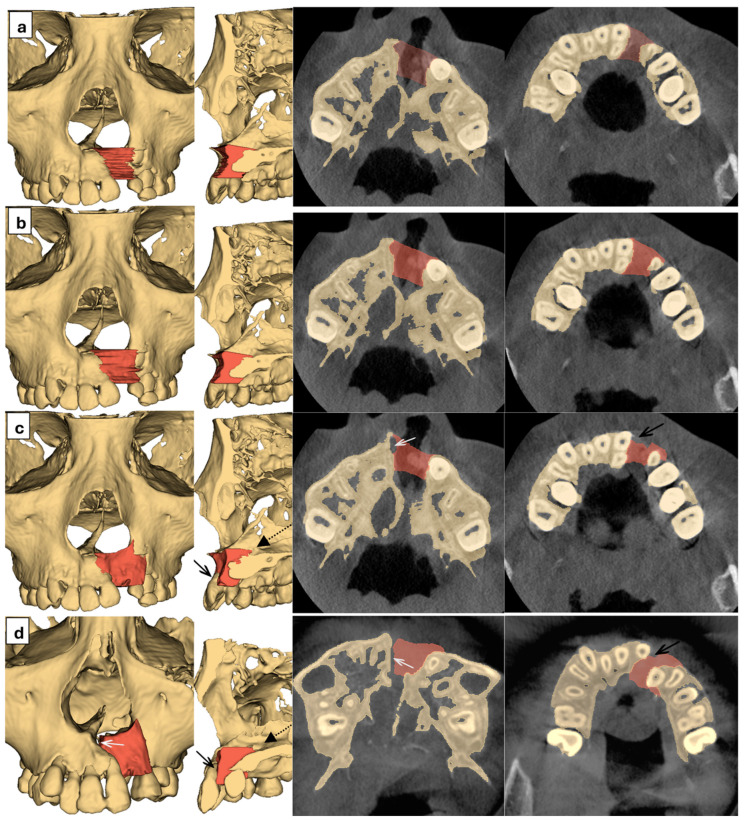
Representative images from Methods A–D displayed as rows from top to bottom (**a**–**d**). Columns from left to right are 3D reconstructions from a frontal view, 3D reconstructions of cross-sections from a lateral view, sliced axial images near ANS height, and sliced axial images near CEJ height, respectively. Shaded areas (Red areas) are the planned graft. Black dotted arrows point to a step between filling and palatal bone surface. Black solid arrows point to a deficiency in defect filling near adjacent teeth. White solid arrows point to a gap between a planned graft and medial bone surface.

Qualitatively, 3D and sliced images were reviewed to assess the restored anatomy and the adequacy of the graft. Examples for each digital method from a single patient can be seen in Figure 4. Several trends of visual findings were observed across multiple patients. Method A yielded a heavily staggered filling between each slice. Methods A and B could only create horizontal upper and lower boundaries because the defect fillings were drawn on selected axial slices, while Methods C and D allowed for a more natural shape, especially in the pyriform area. Sagittally, the superior plane in Methods A and B was placed obliquely towards the upper surface of hard palate, which allowed for more seamless connection with the palate. On the contrary, the posterior border in Methods C and D often had a significant step towards the palatal surface as seen in Figure 4, depending on the nasal floor asymmetry of the patient. Additionally, Methods C and D also often had filling deficiencies in the midline and dental area due to distally deviated ANS and maxillary arch asymmetry. These discrepancies worsened in proportion to the severity of patient asymmetry. In the worst cases in this study, the palatal step was as high as 3.8 mm and the mirrored alveolar segment failed to meet the midline entirely.

## 4. Discussion

To our knowledge, this is the first study explicitly designed to establish a consensus on the boundaries for alveolar bone graft placement, and the most comprehensive study to evaluate four digital and 3D-printed approaches. The consensus of the boundary landmarks was developed between experts from relevant specialties of alveolar bone graft surgery, oral and maxillofacial surgery, orthodontics, and dentomaxillofacial radiology. By comparing multiple methods and approaches, this study provides broader practical insights for radiologic assessment. It was designed to evaluate general workflows rather than promote the capabilities of any single software platform.

This was a simulation study using pliable clay material, which may potentially differ from the compressible particulate bone graft materials commonly used during surgery. Nevertheless, previous studies have reported high correlation and no statistically significant difference between simulated volume and the actual surgical bone volume in alveolar bone graft surgery, which supports the validity for clinical applications [13,19].

Preoperative volume estimation may support surgical decision making, including donor site selection. This may be particularly relevant when the available donor source is limited, such as chin bone [20], or when supplementary allograft material may be needed. These workflows also facilitate the growing application of patient-specific digital design in cleft care, including customized allogenic bone blocks and 3D-printed biomaterials.

Preoperative volumetric determination can also reduce unnecessary risks related to overharvesting of the donor site and under-filling of the defect. Overharvesting increases the risk of complications, including infection, nerve damage, hematoma, fracture, and prolonged hospitalization [15,21]. Inadequate bone bridge formation compromises subsequent orthodontic movement and implant placement, frequently requiring revision surgery. Revision surgery imposes a substantial financial [22] and psychological burden [23] on patients. Therefore, every effort to optimize surgical outcomes is critical. Alveolar bone graft failure rates range from 6% to 44% [24,25]. While success is multifactorial, standardizing bone graft volume represents a modifiable factor that contributes to improved outcomes.

### 4.1. Boundary of Alveolar Defect Filling

The substantial inter-rater discrepancies (up to 0.600 cm^3^) and undesirable reliability in individual operators’ specified plans underscored the need for standardized boundaries. A study by Chou et al. also found moderate reliability (ICC = 0.7) between the filling volume of different surgeons in models [15]. Both the present study and Chou et al. employed a repeated-measures design, allowing assessment of inherent operator variability independent of patient heterogeneity. The most experienced surgeon had the best intra-rater reliability, consistent with other studies [6]. These findings strengthened the original assumption that discrepancies in filling amount may contribute to alveolar bone graft surgery outcome. The orthodontist rater’s graft volume, while not statistically different from either surgeon’s, tended to be lower than that of the most experienced surgeon. It is possible that the orthodontist’s target was biased toward post-resorption bone volume rather than intraoperative filling volume. The variety of the volume obtained formed the basis of reasoning that a consensus of boundary landmarks should be established as a reference standard, instead of relying on a single expert’s opinion.

The findings in the study suggest that explicit definition of boundary landmarks may help in providing consistency between operators of alveolar bone graft surgery. The selected boundaries (ANS, CEJ, incisive canal, alveolar continuity) aligned with the literature’s most cited landmarks while standardizing the previously undefined posterior palatal boundary. Posterior boundary at palatal level (above alveolar level) was very rarely defined in the literature, which could cause inconsistencies. Unlike previous studies that either completely filled palatal defects [13] or used somewhat arbitrary limits [26], we selected the incisive canal as an anatomically recognizable palatal boundary recognizable both clinically and radiographically. The oblique superior plane from the base of the ANS to the hard palate provides a more clinically feasible landmark than the PNS or GPF.

The consensus-derived boundaries demonstrated improved inter- and intra-rater consistency compared to operator-specific plans, suggesting that explicit anatomical landmark definitions can reduce variability in alveolar bone graft planning. These boundaries provide a reproducible framework for both clinical practice and research applications.

### 4.2. Digital Measurement Methods

All digital volumetric measurements were performed using 3D Slicer (www.slicer.org), a general-use and open-source medical image processing software. Even though Kochhar et al. [27] reported no effect of reorientation on defect volume on CBCT, the authors in the study opted for reorientation to account for different head positioning, especially between CT and CBCT. Standardization was implemented by normalizing gray value and uniform voxel size. CT and CBCT have been proven to have equal performance in demonstrating large bony structures such as the maxilla [28,29].

Digital methods will be discussed in the following paragraphs from the least recommended to the most recommended. The undesirable performance of Method C was likely due to the combined challenges of manual landmark placement and sculpting. Previous studies reported inferior reliability of landmark-based superimposition [30], but it was investigated in this study to explore its potential to overcome patient asymmetry. Despite optimizing landmark selection to maximize midline superimposition, inherent craniofacial asymmetry in unilateral clefts such as ANS deviation, nasal floor depression, and alveolar asymmetry prevented the achievement of good anatomy. These asymmetries, potentially more severe in our population, resulted in persistent gaps and deficiencies with superimposition methods.

Mesh-based superimposition, employed in Method D, was established to have better reliability due to automation [30]. It should be noted that both mirroring methods required extensive sculpting to remove structures outside the alveolar bounds, which may introduce substantial operator dependency. A steep learning curve was evident even with trained radiologists. However, additional training would not overcome fundamental issues of asymmetry-induced gaps and filling deficiencies.

Method B yielded acceptable results, both quantitatively and qualitatively. Lower intra-rater reliability, compared to Method A, was likely due to propagation of errors through interpolated slices. Thin, irregular, and rapidly changing structures are more vulnerable to interpolation error. In Method B, this concern is mainly relevant to the buccal and palatal aspects, because the mesial and distal boundaries were defined by subtraction from the maxilla rather than by the operator’s defect segmentation. However, wider clefts may still be associated with greater potential for error because they allow more degrees of freedom along the buccal and palatal contours. However, a high–moderate ICC and easier learning curve suggest potential for optimization through smaller slice intervals or enhanced calibration. One limitation of the method was the abrupt superior/inferior boundaries, which may be undesirable to some operators who may prefer more natural nasal contours, but would still produce an acceptable bone bridge for subsequent dental treatments.

Method A demonstrated the best statistical results in this study. This is a relatively simple workflow that is compatible with many software tools. Abrupt edges and visible stair-stepping on buccal/palatal surfaces may be undesirable, particularly for 3D-printed applications, but may be further addressed with smoothing functions. For volumetric accuracy, Method A remains the most clinically reliable approach [5].

Among the four methods evaluated, axial tracing in every slice demonstrated superior reliability and accuracy, supporting its recommendation. These findings provide empirical guidance for selecting suitable measurement methods based on clinical context and population characteristics.

### 4.3. Implications and Future Work

A volumetric workflow for preoperative alveolar bone graft planning can support several practical objectives. As three-dimensional evaluation becomes increasingly popular, future research is encouraged to adopt standardized protocols to enable meaningful comparisons.

Volumetric evaluation will likely increase with advances in AI. Anatomy-aware automatic segmentation can improve measurement accuracy [31], while predictive algorithms integrating preoperative volume, cleft type, canine status, and orthodontic plans may estimate personalized probabilities of bony bridge success and donor volume requirements [13]. The consensus boundaries and validated methods established here provide a foundation for developing more consistent, efficient, and reproducible AI-assisted planning tools.

Digital and physical simulations can also be used as training devices to improve surgical performance or education [32,33]. Digital workflows also enable the design and fabrication of customized devices, including 3D-printed molds, 3D-printed biomaterials, scaffolds, and custom allogenic bone blocks to improve bony formation [14,34]. Validated design protocols ensure fit, stability, and reproducibility. These digital simulations can also be further extended to digital virtual surgery for cleft patients.

### 4.4. Strengths and Limitations

Strengths of this study included establishing a consensus from multiple specialties involved in alveolar bone graft surgery with comprehensive comparisons between multiple digital methods and a physical reference standard. This study incorporated qualitative assessments that supplemented quantitative analysis with a strong emphasis on clinical applicability. These visual evaluations revealed clinically relevant limitations that volumetric statistics alone could not capture. The use of physical models allowed for the comparison of different boundaries in the same patient with intra- and inter-rater evaluations. Careful methodology was applied to the 3D-printed models by using established practices to ensure accuracy, and dimensional deviations of the models were quantified. The clay volume was determined by weight (mass measured to 0.0001 g), providing substantially higher precision than the water-displacement approaches commonly reported in prior studies.

Limitations included the use of datasets from various CT and CBCT machines. Standardization of voxel sizes, gray values, and patient orientation were conducted to minimize variability. This study had a small sample size from a single center, which limits statistical power. Participants in this study tended to have more severe asymmetry; however, this study provided valuable data points regarding alveolar bone graft planning in patients with more severe presentations. Larger studies incorporating more cleft types, a larger sample size, and multiple cleft centers across diverse populations are recommended. Clinical validation is needed to correlate preoperative volumetric planning with actual surgical volumes and postoperative outcomes.

## 5. Conclusions

This study established protocols for the volumetric assessment of alveolar cleft defects, addressing two critical gaps in preoperative planning: boundary landmark definitions and measurement methodology validation. Standardized anatomical boundaries were defined as a tilted plane connecting the base of the ANS to the hard palate superiorly, CEJ inferiorly, incisive canal as the posterior palatal limit, and alveolar continuity laterally, with symmetrical buccal and palatal contours. Volumetric measurement with axial tracing in every slice using standardized boundary definitions demonstrated robust accuracy and reliability. This validated measurement protocol provides a foundation for more consistent surgical planning between operators and enables meaningful cross-study comparisons in alveolar bone graft research.

## Figures and Tables

**Table 1:** Mean defect volumes, intra-rater, and inter-rater ICC of digital measurements. (A, excellent; B, good; C, moderate; D, poor.)

Measurement	Reference Standard	Method A		Method B		Method C		Method D	
		Rater 1	Rater 2	Rater 1	Rater 2	Rater 1	Rater 2	Rater 1	Rater 2
Mean defect volume	1.280 ± 0.241	1.254 ± 0.385	1.281 ± 0.379	1.271 ± 0.325	1.274 ± 0.307	1.287 ± 0.292	0.803 ± 0.378	1.167 ± 0.436	0.989 ± 0.333
Intra-rater ICC	Not applicable	0.991 ^A^	0.971 ^A^	0.883 ^B^	0.951 ^A^	0.691 ^C^	0.919 ^A^	0.924 ^A^	0.977 ^A^
Inter-rater ICC	Not applicable	0.965 ^A^		0.921 ^A^		0.017 ^D^		0.323 ^D^	

## Data Availability

The original contributions presented in this study are included in the article. Further inquiries can be directed to the corresponding author.
